# Supplementary material for: A SIRT7-dependent acetylation switch regulates early B cell differentiation and lineage commitment through Pax5
Source: Nat Immunol. 2024 Oct 18;25(12):2308–19. doi: 10.1038/s41590-024-01995-7 (PMC11588656; doi:10.1038/s41590-024-01995-7)
Supplement: Supplementary file 1 — Reporting Summary [file 41590_2024_1995_MOESM1_ESM.pdf]

nature portfolio

Corresponding author(s): Alejandro Vaquero
Berta N Vazquez

Last updated by author(s): Sep 19, 2024

Reporting Summary

Nature Portfolio wishes to improve the reproducibility of the work that we publish. This form provides structure for consistency and transparency in reporting. For further information on Nature Portfolio policies, see our Editorial Policies and the Editorial Policy Checklist.

Statistics

For all statistical analyses, confirm that the following items are present in the figure legend, table legend, main text, or Methods section.

n/a	Confirmed	
☐	☒	The exact sample size (*n*) for each experimental group/condition, given as a discrete number and unit of measurement
☐	☒	A statement on whether measurements were taken from distinct samples or whether the same sample was measured repeatedly
☐	☒	The statistical test(s) used AND whether they are one- or two-sided<br>*Only common tests should be described solely by name; describe more complex techniques in the Methods section.*
☒	☐	A description of all covariates tested
☐	☒	A description of any assumptions or corrections, such as tests of normality and adjustment for multiple comparisons
☐	☒	A full description of the statistical parameters including central tendency (e.g. means) or other basic estimates (e.g. regression coefficient) AND variation (e.g. standard deviation) or associated estimates of uncertainty (e.g. confidence intervals)
☐	☒	For null hypothesis testing, the test statistic (e.g. *F*, *t*, *r*) with confidence intervals, effect sizes, degrees of freedom and *P* value noted<br>*Give P values as exact values whenever suitable.*
☒	☐	For Bayesian analysis, information on the choice of priors and Markov chain Monte Carlo settings
☒	☐	For hierarchical and complex designs, identification of the appropriate level for tests and full reporting of outcomes
☐	☒	Estimates of effect sizes (e.g. Cohen's *d*, Pearson's *r*), indicating how they were calculated

Our web collection on statistics for biologists contains articles on many of the points above.

Software and code

Policy information about availability of computer code

Data collection	Flow cytometry samples were run in a FACS Canto II (BD Biosciences) or sorted with a FACS Aria II (BD Biosciences). Data were collected with BD FACS Diva software 6.1.3.<br>Western blot images were obtained with an iBright 1500 (Invitrogen).<br>ELISA results were read in a Multiskan Sky (ThermoFisher) plate reader.<br>Gel filtration HPLC were performed with a GE AKTA Purifier 10 FPLC System.<br>RT-qPCR experiments were run on a QuantStudio 5 (ThermoFisher).<br>Chromatin shearing for ChIP experiments was performed with a Covaris M220 system.<br>Mass spectrometry data were acquired on an Orbitrap Fusion Lumos Tribrid system (ThermoFisher).<br>ChIP-Seq and RNA-Seq data were acquired on a DNBSEQ-G400 instrument.
Data analysis	Data presentation and statistical analyses: GraphPad Prism 8.0.1., ggplot 3.5.1. and Microsoft Excel 2309.<br>RT-qPCR data were processed with QuantStudio Design & Analysis Software 1.5.1.<br>Flow cytometry data analysis: FlowJo 7.6.<br>Western blot densitometric estimation and histological sections processing: ImageJ 1.52a.<br>Mass spectrometry data were processed with Xcalibur software (Proteome, version 4.0.27.10; PAX5 acetylome, version 4.2.28.14) (ThermoFisher).<br>sc-RNA-Seq visualization and data collection: Single Cell Portal (Broad Institute).<br>Gene set enrichment analysis: GSEA 4.1.0.<br>Heatmap visualization and clustering: Morpheus software (Broad Institute).<br>Gene ontolgy terms: Enrichr tool.<br>Visualization of ChIP-Seq and RNA-Seq tracks: Integrated Genome Viewer 2.7.2.

Motif Analysis: HOMER v.5.0.1.
Differential Expression Analysis: DESeq2
ChIP-Seq binding profiles: DiffBind and plotHeatmap (deepTools v3.5.1)

For manuscripts utilizing custom algorithms or software that are central to the research but not yet described in published literature, software must be made available to editors and reviewers. We strongly encourage code deposition in a community repository (e.g. GitHub). See the Nature Portfolio guidelines for submitting code & software for further information.

Data

Policy information about availability of data

All manuscripts must include a data availability statement. This statement should provide the following information, where applicable:

- Accession codes, unique identifiers, or web links for publicly available datasets
- A description of any restrictions on data availability
- For clinical datasets or third party data, please ensure that the statement adheres to our policy

All Pax5WT, Pax5K198Q and Pax5K198R ChIP-Seq data and RNA-Seq data from wild-type and Sirt7-/- bone marrow pro-B and pre-B cells; Pax5-/- B cell progenitors retrovirally expressing EV, Pax5WT, Pax5K198Q and Pax5K198R; and wild-type or Sirt7-/- B cell progenitors expressing EV, Pax5WT or Pax5K198R have been deposited at the National Center for Biotechnology Information Gene Expression Omnibus (GEO) and are available under the accession number: GSE246370. All sequencing data were aligned onto the mm10 reference mouse genome. Proteomics data from wild-type and Sirt7-/- bone marrow pre-B cells and Pax5 acetylation experiments have been deposited to the ProteomeXchange Consortium via the PRIDE partner repository with the dataset identifier PXD046457.

Research involving human participants, their data, or biological material

Policy information about studies with human participants or human data. See also policy information about sex, gender (identity/presentation), and sexual orientation and race, ethnicity and racism.

Reporting on sex and gender	N/A
Reporting on race, ethnicity, or other socially relevant groupings	N/A
Population characteristics	N/A
Recruitment	N/A
Ethics oversight	N/A

Note that full information on the approval of the study protocol must also be provided in the manuscript.

Field-specific reporting

Please select the one below that is the best fit for your research. If you are not sure, read the appropriate sections before making your selection.

☒ Life sciences ☐ Behavioural & social sciences ☐ Ecological, evolutionary & environmental sciences

For a reference copy of the document with all sections, see nature.com/documents/nr-reporting-summary-flat.pdf

Life sciences study design

All studies must disclose on these points even when the disclosure is negative.

Sample size	No sample size calculation was performed, as experiments were performed on genetically identical mice or cell lines. Sample size was based on previous experiments, as well as on the 3R principales to reduce the number of animal used. Sample sizes were sufficient to detect differences between experimental groups.
Data exclusions	No data was excluded from the manuscript.
Replication	All replicates were performed under the same conditions and are described in the manuscript. All attempts of replication were successful. Results were similarly replicated in at least two independent experiments, and results pooled from independent experiments are indicated in the corresponding figure legends.
Randomization	For in vivo transplantation experiments, recipient mice were randomized to exclude age- and sex-related effects. The rest of the experiments were performed with genetically identical mice, so randomization was not required.
Blinding	Investigators were not blinded, as most experiments were performed by a single person. In the case of sequencing and proteomics experiments, much of the bioinformatic analysis was performed by an independent person that was not involved in the performance of the experiment.

Reporting for specific materials, systems and methods

We require information from authors about some types of materials, experimental systems and methods used in many studies. Here, indicate whether each material, system or method listed is relevant to your study. If you are not sure if a list item applies to your research, read the appropriate section before selecting a response.

Materials & experimental systems

n/a	Involved in the study
☐	☒ Antibodies
☐	☒ Eukaryotic cell lines
☒	☐ Palaeontology and archaeology
☐	☒ Animals and other organisms
☒	☐ Clinical data
☒	☐ Dual use research of concern
☒	☐ Plants

Methods

n/a	Involved in the study
☐	☒ ChIP-seq
☐	☒ Flow cytometry
☒	☐ MRI-based neuroimaging

Antibodies

Antibodies used

For flow cytometry, all antibodies were used at a dilution of 1:400, unless otherwise specified:
anti-B220 (RA3-6B2, eBioscience, 17-0452-81)
anti-CD19 (HIB19, eBioscience, 562440)
anti-CD43 (eBioR2/60, eBioscience, 1:800 dilution, 11-0431-82)
anti-CD43 (1510, BD Biosciences, 1:800 dilution, 563377)
anti-IgM (II/41, eBioscience, 1:800 dilution, 553437)
anti-IgD (11-26c, eBioscience, 47-5993-80)
anti-CD21 (7G6, BDBiosciences, 1:800 dilution, 552957)
anti-CD23 (B3B4, BDBiosciences, 1:200 dilution, 553137)
anti-CD93 (AA4.1, eBioscience, 11-5892-82)
anti-GL7 (GL-7, eBioscience, 1:200 dilution, 48-5902-82)
anti-CD38 (90, eBioscience, 406-0381-80)
anti-CD138 (300506, Invitrogen, MA5-23553)
anti-Fas (SA367H8, Biolegend, 1:600 dilution, 152617)
anti-IgG1 (A85-1, BD Pharmigen, 1:600 dilution, 553441)
anti-CD127 (eBioSD/199, eBioscience, 1:200 dilution, 12-1273-81)
anti-CD45.1 (A20, eBioscience, 11-0453-81)
anti-CD45.2 (104, eBioscience, 1:200 dilution, 17-0454-81)
anti-TCRβ (H57-597, BD Biosciences, 1:200 dilution, 14-5961-82)
anti-NKp46 (29A1.4, Biolegend, 1:100 dilution, 137618)
anti-CD4 (GSK1.5, eBioscience, 1:800 dilution, 46-0041-80)
anti-CD8 (53-6.7, eBioscience, 1:800 dilution, 12-0081-81)
anti-hCD4 (RPA-T4, Biolegend, 300504)
anti-CD3e (145-2C11, BD Biosciences, 559971)
anti-Ly76 (TER-119, BD Biosciences, 51-09082J)
anti-CD11b (M1/70, BD Biosciences, 51-01712J)
anti-Gr1 (RB6-8C5, BD Biosciences, 51-01212J)
anti-PAX5 (1H9, eBioscience, 0.2 µg per sample, 14-9918-82)
anti-SIRT7 (D3K5A, Cell signaling, 0.25 µg per sample, 5360)
anti-STAT5-pY694 (47/Stat5(pY694), 20 µL per sample, BD Biosciences, 612567)
anti-Rabbit IgG (H+L) polyclonal secondary antibody (Invitrogen, 0.25 µg per sample, 11034)

For magnetic purification of CD19+ cells:
anti-CD19 (1D3, BD Biosciences, 0.1 µg per 10^6 cells, 553784)

For ChIP-Seq:
anti-PAX5 (Abcam, 10 µg per sample, ab183575)

For Western Blot, all antibodies were used at a dilution of 1:1000 in PBS-0.1% Tween, unless otherwise specified:
anti-SIRT7 (D3K5A, Cell signaling, 5360S)
anti-PAX5 (D19F8, Cell signaling, 8970S)
anti-H3 (ab1791, Abcam)
anti-Fibrillarin (B1, Santa Cruz Biotechnology, sc-166001)
anti-Acetyl-lysine (Ac-K2-100, Cell signaling, 1:200 dilution, 9814)
anti-FLAG (M2, Sigma-Aldrich, 1:10,000 dilution, A8592)
anti-HA (Sigma, 1:5,000 dilution, H6908)
anti-Myc-Tag (9B11, Cell signaling, 2276)
anti-V5 (ab9116, Abcam)
anti-Actin (AC-15, Sigma-Aldrich, 1:5000 dilution, A1978)

For ELISA:
rat anti-mouse IgM (Ig Isotyping Mouse Uncoated ELISA Kit, Invitrogen, 1/250 dilution, 88-50630)
rat anti-mouse IgG1 (Ig Isotyping Mouse Uncoated ELISA Kit, Invitrogen, 1/250 dilution, 88-50630)
rat anti-mouse IgG3 (Ig Isotyping Mouse Uncoated ELISA Kit, Invitrogen, 1/250 dilution, 88-50630)
goat anti-rat IgG conjugated to HRP (Sigma-Aldrich, 1:5000 dilution, AP136P)

For immunoprecipitation:
anti-FLAG (M2, Sigma, 20ul per sample, A2220)

Validation

All the antibodies used are commercially available and were validated by the manufacturer for the intended applications and species (validation information available at vendor's website), with one exception: the anti-SIRT7 (D3K5A, Cell signaling) antibody has not been reported for flow cytometry, but we have validated it in our lab using proper isotype controls as well as wild-type, SIRT7-deficient, and SIRT7-overexpressing mouse cells to determine its specificity.

Eukaryotic cell lines

Policy information about cell lines and Sex and Gender in Research

Cell line source(s)

KOPN-8, NALM-20, REH, TANOUE, SD-1, TOM-1 and SEM cells were purchased from the DSMZ-German Collection of Microorganisms and Cell Cultures GmbH and were kindly provided by M. Parra (IJC, Barcelona, Spain). HAFTL cells are a fetal liver-derived, Ha-Ras-transformed mouse pre-B cell line that have been previously described (Alessandrini A. et al. Continuing rearrangement of immunoglobulin and T-cell receptor genes in a Ha-ras-transformed lymphoid progenitor cell line.Proc Natl Acad Sci U S A 84(7): 1799–1803 (1987) and were provided by Dr. Maribel Parra (IJC, Barcelona, Spain). OP9, HEK293F cells were purchased from the American Type Culture Collection (ATCC). Platinum E cells were purchased from Cell Biolabs. Primary pro-B cells were obtained as detailed in the Methods section from both male and female mice.

Authentication

Cell lines were not authenticated.

Mycoplasma contamination

All tested cell lines were negative for mycoplasma contamination.

Commonly misidentified lines
(See ICLAC register)

None of the cell lines used were found in the Commonly misidentified lines database

Animals and other research organisms

Policy information about studies involving animals; ARRIVE guidelines recommended for reporting animal research, and Sex and Gender in Research

Laboratory animals

Wild-type, Sirt7-/- and IgHEL+ mice were in the 129Sv background, unless otherwise specified in the manuscript. In these cases, C57BL/6 wild-type and Sirt7-/- were used to confirm our observations on a different background.
Pax5-/- and CD45.2 mice were in the C57BL/6 background. Heterozygous CD45.1+CD45.2+ mice were generated by crossing C57BL/6 CD45.1 and Wt 129Sv CD45.2 mice for one generation. Both sexes were included in all experiments, and only mice of 8-16 weeks were used, except for isolation of Pax5-/- progenitors, that were obtained from murine fetal livers.

Wild animals

Study did not involve wild animals.

Reporting on sex

Both sexes were included in all experiments, and no sex comparisons have been performed.

Field-collected samples

Study did not involve field-collected samples.

Ethics oversight

Animal studies were conducted at IJC (Spain) according to national authorities and institutional ethics committees (Germans Trias i Pujol Reserach Institute Ethics Committee). The collection of bone marrow samples from C57BL/6 wild-type and Sirt7-/- mice, and the generation of Pax5-/- mouse B cell progenitors were conducted according to national authorities and institutional ethics committees at MPI-HLR (Germany) and Lund University (Sweden), respectively.

Note that full information on the approval of the study protocol must also be provided in the manuscript.

Plants

Seed stocks

Report on the source of all seed stocks or other plant material used. If applicable, state the seed stock centre and catalogue number. If plant specimens were collected from the field, describe the collection location, date and sampling procedures.

Novel plant genotypes

Describe the methods by which all novel plant genotypes were produced. This includes those generated by transgenic approaches, gene editing, chemical/radiation-based mutagenesis and hybridization. For transgenic lines, describe the transformation method, the number of independent lines analyzed and the generation upon which experiments were performed. For gene-edited lines, describe the editor used, the endogenous sequence targeted for editing, the targeting guide RNA sequence (if applicable) and how the editor was applied.

Authentication

Describe any authentication procedures for each seed stock used or novel genotype generated. Describe any experiments used to assess the effect of a mutation and, where applicable, how potential secondary effects (e.g. second site T-DNA insertions, mosiacism, off-target gene editing) were examined.

ChIP-seq

Data deposition

☒ Confirm that both raw and final processed data have been deposited in a public database such as GEO.

☒ Confirm that you have deposited or provided access to graph files (e.g. BED files) for the called peaks.

Data access links<br>*May remain private before publication.*	ChIP-Seq data are available at GEO under the accession number: GSE246370 (https://www.ncbi.nlm.nih.gov/geo/query/acc.cgi?acc=GSE246370)
Files in database submission	GSM7867743 Wt_proB_1<br>GSM7867744 Wt_preB_1<br>GSM7867745 Sirt7-/-_proB_1<br>GSM7867746 Sirt7-/-_preB_1<br>GSM7867747 Wt_proB_2<br>GSM7867748 Wt_preB_2<br>GSM7867749 Sirt7-/-_proB_2<br>GSM7867750 Sirt7-/-_preB_2<br>GSM7867751 Pax5_EV_1_RNA<br>GSM7867752 Pax5_EV_2_RNA<br>GSM7867753 Pax5_Wt_1_RNA<br>GSM7867754 Pax5_Wt_2_RNA<br>GSM7867755 Pax5_K198Q_1_RNA<br>GSM7867756 Pax5_K198Q_2_RNA<br>GSM7867757 Pax5_K198R_1_RNA<br>GSM7867758 Pax5_K198R_2_RNA<br>GSM8376251 Input_Pax5_Wt_1<br>GSM8376252 Input_Pax5_Wt_2<br>GSM8376253 Input_Pax5_K198Q_1<br>GSM8376254 Input_Pax5_K198Q_2<br>GSM8376255 Input_Pax5_K198R_1<br>GSM8376256 Input_Pax5_K198R_2<br>GSM8376257 ChIP_Pax5_Wt_1<br>GSM8376258 ChIP_Pax5_Wt_2<br>GSM8376259 ChIP_Pax5_K198Q_1<br>GSM8376260 ChIP_Pax5_K198Q_2<br>GSM8376261 ChIP_Pax5_K198R_1<br>GSM8376262 ChIP_Pax5_K198R_2<br>GSM8376263 WT_EV_1<br>GSM8376264 WT_EV_2<br>GSM8376265 Sirt7-/-_EV_1<br>GSM8376266 Sirt7-/-_EV_2<br>GSM8376267 Sirt7-/-_Pax5Wt_1<br>GSM8376268 Sirt7-/-_Pax5Wt_2<br>GSM8376269 Sirt7-/-_Pax5K198R_1<br>GSM8376270 Sirt7-/-_Pax5K198R_2
Genome browser session<br>(e.g. UCSC)	*Provide a link to an anonymized genome browser session for "Initial submission" and "Revised version" documents only, to enable peer review. Write "no longer applicable" for "Final submission" documents.*

Methodology

Replicates	Two biological replicates for each sample.
Sequencing depth	Clean reads per sample (SE50 sequencing):<br>Input_Pax5_Wt_1: 25,309,841<br>Input_Pax5_Wt_2: 25,294,390<br>Input_Pax5_K198Q_1: 25,291,043<br>Input_Pax5_K198Q_2: 25,280,864<br>Input_Pax5_K198R_1: 25,271,600<br>Input_Pax5_K198R_2: 25,308,898<br>ChIP_Pax5_Wt_1: 25,214,352<br>ChIP_Pax5_Wt_2: 25,271,937<br>ChIP_Pax5_K198Q_1: 25,174,210<br>ChIP_Pax5_K198Q_2: 25,201,975<br>ChIP_Pax5_K198R_1: 25,205,736<br>ChIP_Pax5_K198R_2: 25,209,517
Antibodies	anti-PAX5 (Abcam, 10 μg per sample, ab183575, Lot #1037689-1)
Peak calling parameters	Peak calling was performed using MACS2 "callpeak" (-f BAM --nomodel --extsize 20 -g mm -B) taking into account both IP (-t) and input (-c) samples for each peak calling.

Data quality	All the raw .fastq files were quality checked using FastQC. Universal adapters were trimmed using Trim Galore. After Bowtie2 alignment, we selected from the sorted BAM files only those reads that were succesfully aligned to the genome, eliminating all th.e PCR duplicates and filtering reads by MAPQ > 36 using SAMtools and SAMbamba. BigWig files were generated using DeepTools
Software	Windows 10 with "Ubuntu on Windows" (v22.04.2 LTS), with LAN access to our High-Performance Computer (HPC) server (Josep Carreras Leukaemia Research Institute supercomputer). Ubuntu terminal using Bash as standard language with all the associated packages retrieved from our internal HPC server (Bowtie2, STAR, MACS2, Salmon, SAMbamba, SAMtools, FastQC, Trim Galore, plotHeatmap, DeepTools). R-Studio for using R-Language and associated packages (Tximeta, DiffBind, ggplot2, DESeq2, ChIPpeakAnno). Anaconda3 "Conda" environment to execute Python associated packages (Scanpy).

Flow Cytometry

Plots

Confirm that:

☒ The axis labels state the marker and fluorochrome used (e.g. CD4-FITC).

☒ The axis scales are clearly visible. Include numbers along axes only for bottom left plot of group (a 'group' is an analysis of identical markers).

☒ All plots are contour plots with outliers or pseudocolor plots.

☒ A numerical value for number of cells or percentage (with statistics) is provided.

Methodology

Sample preparation	Bone marrow, spleen and thymus samples were collected from wild-type, Sirt7-/-, IgHEL+, Sirt7-/-IgHEL+, CD45.1+CD45.2+ and CD45.2+ mice. Bone marrow samples were crushed in staining buffer (3% fetal bovine serum (FBS), 2 mM EDTA in phosphate buffer saline (PBS)) to obtain single cell suspensions. Spleen and thymus samples were similarly processed. Red blood cells were lysed in ACK buffer (Gibco), and the reaction was stopped by adding five volumes of staining buffer. Cells were filtered through 40-µm sterile strainers and incubated with Fc-block (eBioscience) before staining for 30 min on ice with the corresponding antibodies. Stained cells were then washed and analyzed or sorted.
Instrument	FACS Canto II and LSRFortessa SORP (BD Biosciences).
Software	Data were collected with BD FACS Diva software 6.1.3. and analyzed with FlowJo 7.6. sofware.
Cell population abundance	Sorted populations were re-run on a flow cytometer to ensure their purity.
Gating strategy	In all samples, singlets were gated first by FSC-H and FSC-A. Progenitors/Lymphocytes were subsequently gated by FSC VS SSC.

☒ Tick this box to confirm that a figure exemplifying the gating strategy is provided in the Supplementary Information.
