# Supplementary figures and images for: A SIRT7-dependent acetylation switch regulates early B cell differentiation and lineage commitment through Pax5
Source: Nat Immunol. 2024 Oct 18;25(12):2308–19. doi: 10.1038/s41590-024-01995-7 (PMC11588656; doi:10.1038/s41590-024-01995-7)

**b**

SIRT7

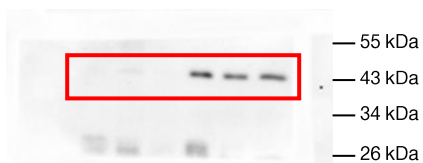

H3

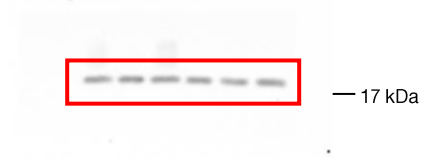

Supplement: Supplementary file 9 — Unprocessed western blots and/or gels. [file 41590_2024_1995_MOESM9_ESM.pdf]

**b**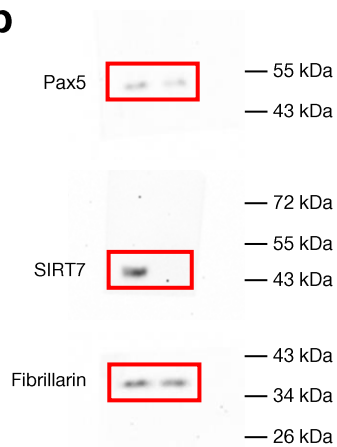**f**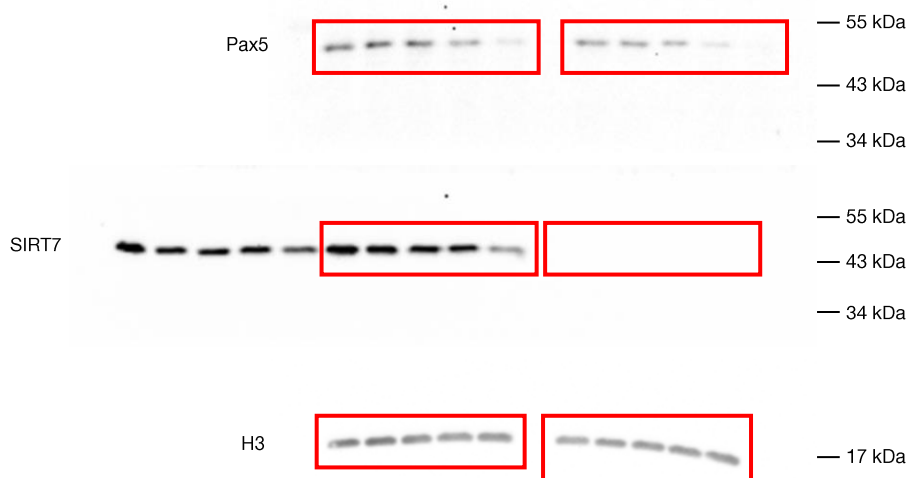**d**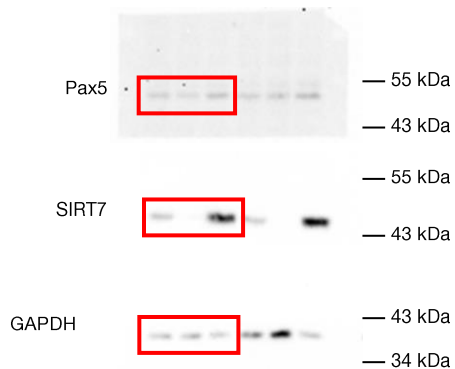**i**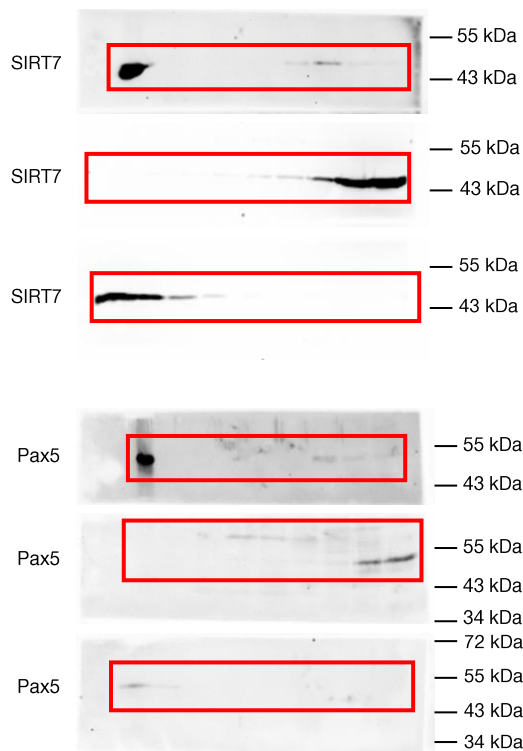**h**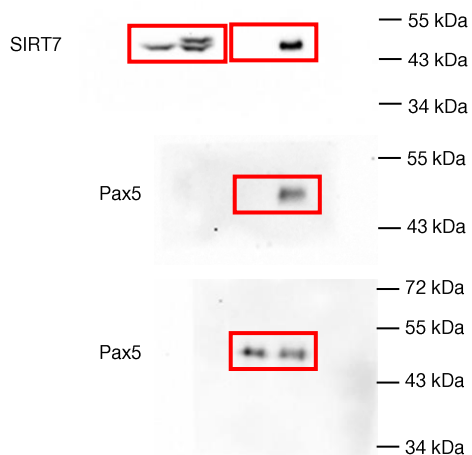**j**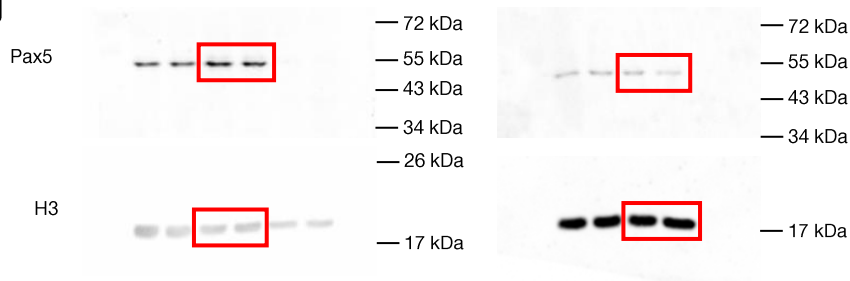

Supplement: Supplementary file 12 — Unprocessed western blots and/or gels. [file 41590_2024_1995_MOESM12_ESM.pdf]

**a**

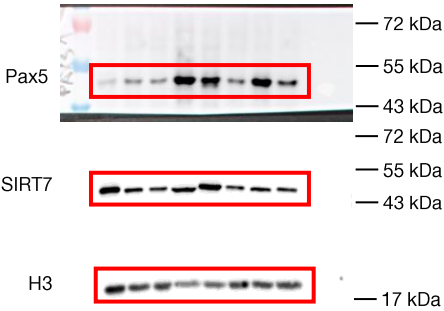

**d**

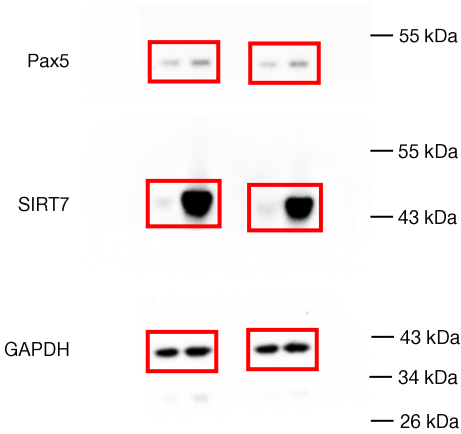

Supplement: Supplementary file 15 — Unprocessed western blots and/or gels. [file 41590_2024_1995_MOESM15_ESM.pdf]

**e**

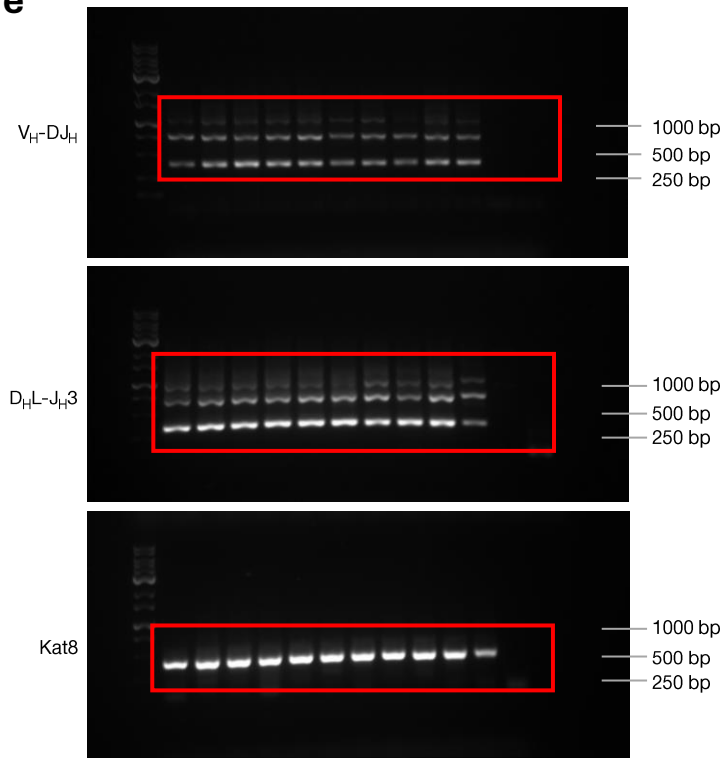

Supplement: Supplementary file 19 — Unprocessed western blots and/or gels. [file 41590_2024_1995_MOESM19_ESM.pdf]

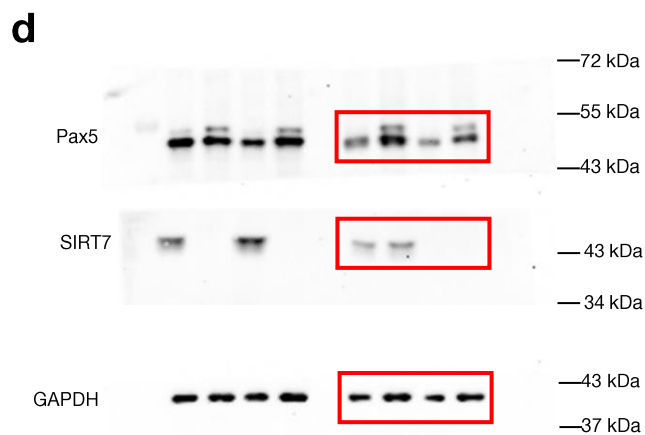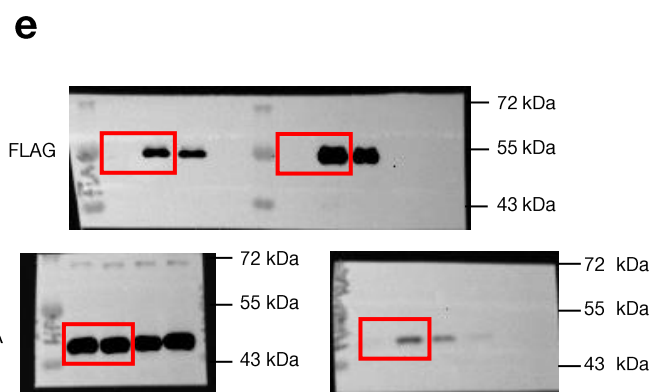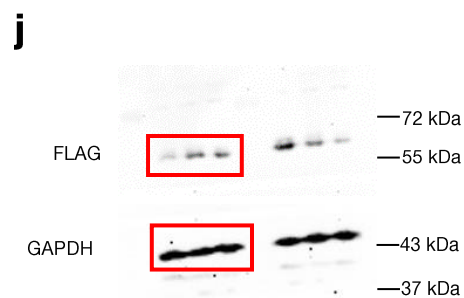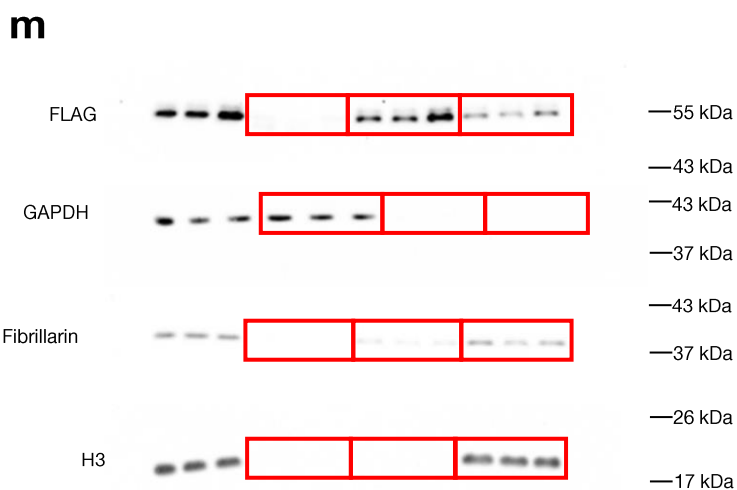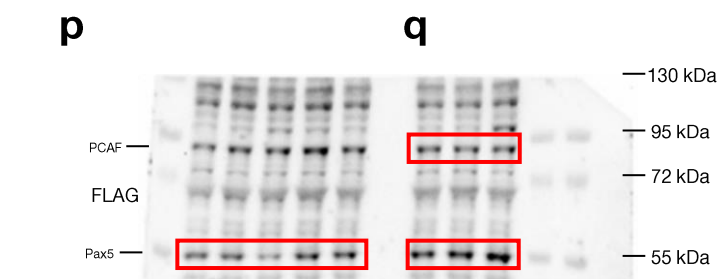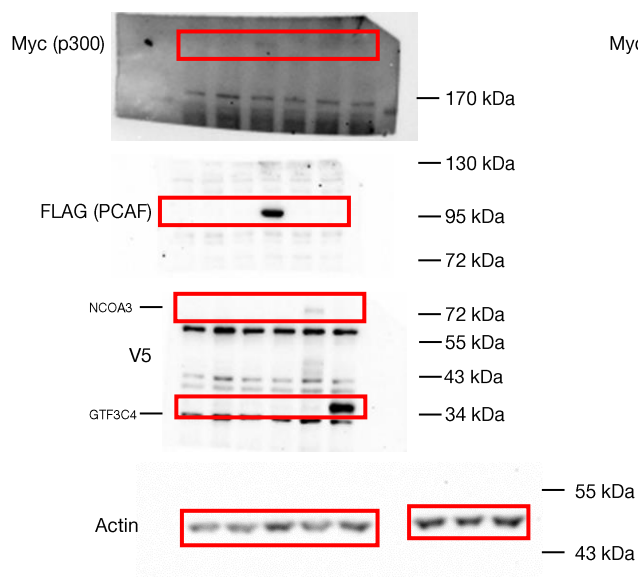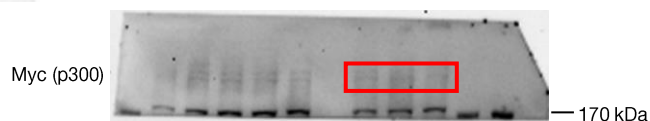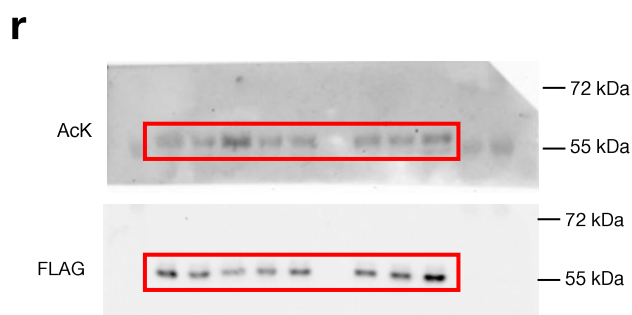

Supplement: Supplementary file 21 — Unprocessed western blots and/or gels. [file 41590_2024_1995_MOESM21_ESM.pdf]
